# Dark Tetrad Traits and Suicidal Ideation in Psychiatric Patients: The Role of Depression, Anxiety, and Stress

**DOI:** 10.3390/bs16091606

**Published:** 2026-09-09

**Authors:** Marin Mamić, Nikolina Farčić, Ivana Barać, Željko Mudri, Marija Barišić, Blaženka Kljaić Bukvić, Ivana Mamić, Zrinka Puharić, Ivan Vukoja

**Affiliations:** 1 General County Hospital Požega, Osječka 107, 34000 Požega, Croatia; ivana.mamic@pozeska-bolnica.hr (I.M.); ivan.vukoja@pozeska-bolnica.hr (I.V.); 2 Faculty of Dental Medicine and Health Osijek, Josip Juraj Strossmayer University of Osijek, Crkvena 21, 31000 Osijek, Croatia; nfarcic@fdmz.hr (N.F.); ibarac@fdmz.hr (I.B.); zmudri@fdmz.hr (Ž.M.); mbarisic@fdmz.hr (M.B.); bkbukvic@fdmz.hr (B.K.B.); zpuharic@vub.hr (Z.P.); 3 Department of Neurosurgery, University Hospital Centre Osijek, Josip Huttler Street 4, 31000 Osijek, Croatia; 4 Department of Nursing, Bjelovar University of Applied Sciences, Trg Eugena Kvaternika 4, 43000 Bjelovar, Croatia; 5 Faculty of Medicine, Josip Juraj Strossmayer University of Osijek, J. Huttlera 4, 31000 Osijek, Croatia; 6 Faculty of Medicine, University of Rijeka, Braće Branchetta 20, 51000 Rijeka, Croatia

**Keywords:** Dark Tetrad, sadism, suicidal ideation, depression, anxiety, stress, psychiatric patients, indirect effects

## Abstract

This study examined direct and indirect associations of Machiavellianism, narcissism, psychopathy, and sadism with suicidal ideation through depression, anxiety, and stress in psychiatric patients. A cross-sectional sample of 210 patients completed the Short Dark Tetrad (SD4), Depression Anxiety Stress Scales-21 (DASS-21), and Suicidal Ideation Attributes Scale (SIDAS). Regression paths were estimated using robust maximum likelihood, whereas indirect effects were evaluated using 10,000 bootstrap resamples. Because 92 participants (43.8%) had SIDAS = 0, two-part sensitivity analyses separated the occurrence from the severity of suicidal ideation; additional analyses excluded the two smallest diagnostic groups. Depression, stress, and anxiety correlated with SIDAS (r = 0.560, 0.509, and 0.466, respectively). The sadism–stress–SIDAS indirect effect was significant in the primary model (B = 0.147; 95% bootstrap CI [0.037, 0.291]), after covariate adjustment (B = 0.126; 95% bootstrap CI [0.016, 0.266]), and after excluding psychotic and bipolar groups (B = 0.115; 95% CI [0.006, 0.259]). In two-part analyses, depression was associated with SIDAS occurrence, whereas stress and sadism were associated with severity among participants with SIDAS > 0. The findings support a stress-related indirect association between sadism and suicidal ideation, but causal interpretation is not warranted.

## 1. Introduction

Suicidal thoughts and behaviors represent some of the most serious clinical outcomes in psychiatry. Suicidality is not a unitary phenomenon but encompasses suicidal ideation, planning, attempts, and death by suicide, and the factors associated with the emergence of suicidal thoughts are not necessarily the same as those associated with the transition from ideation to behavior (Klonsky & May, 2015; Klonsky et al., 2018; O’Connor & Kirtley, 2018; Turecki et al., 2019). Contemporary models therefore emphasize the distinction between distal vulnerabilities and proximal psychological processes that may increase current risk.

The theoretical framework of this study draws on the stress-diathesis approach, the Integrated Motivational-Volitional (IMV) model, and interpersonal conceptualizations of suicidality (Mann et al., 1999; O’Connor & Kirtley, 2018; Van Orden et al., 2010). The stress-diathesis model proposes that relatively stable individual vulnerability interacts with acute or chronic stressors. The IMV model distinguishes background factors and vulnerabilities from motivational processes leading to suicidal ideation and volitional factors facilitating the transition to suicidal behavior. The interpersonal theory emphasizes thwarted belongingness and perceived burdensomeness. A common implication of these approaches is that suicidal ideation rarely arises from a single isolated characteristic. The IMV model is used here as a conceptual framework rather than as a model directly tested in this study, because the DASS-21 does not operationalize defeat, entrapment, or other specific motivational constructs proposed by the IMV model.

Personality traits may represent a distal layer of such vulnerability. Particular attention has been given to socially aversive traits grouped under the Dark Triad—Machiavellianism, narcissism, and psychopathy (Furnham et al., 2013; Paulhus & Williams, 2002; Vize et al., 2018). The Dark Tetrad extends this framework by including everyday sadism (Buckels et al., 2013; O’Meara et al., 2011; Paulhus et al., 2021). Machiavellianism is characterized by strategic manipulation, cynicism, and instrumental use of others; narcissism by grandiosity, entitlement, and a need for validation; psychopathy by callousness, reduced empathy, and impulsivity; and sadism by a tendency to derive pleasure from cruelty or the suffering of others (Buckels et al., 2013; Jones & Paulhus, 2014; Muris et al., 2017; O’Meara et al., 2011; Paulhus et al., 2021).

The literature on dark traits has focused predominantly on externalizing and interpersonal outcomes, including aggression, antisocial behavior, manipulation, trolling, and other socially harmful behaviors (Buckels et al., 2013; Jones & Paulhus, 2014; Muris et al., 2017; O’Meara et al., 2011; Paulhus et al., 2021). Empirical evidence directly connecting dark traits with suicidality remains comparatively limited, but several studies suggest clinically relevant associations. In a community sample of high-school students, Chabrol et al. (2015) identified a high Dark Tetrad profile characterized by the highest levels of both antisocial behavior and suicidal ideation. Harrop et al. (2017) further showed that specific facets of psychopathy and pathological narcissism were associated with components of the interpersonal-psychological theory of suicide across community, undergraduate, and military samples. In a two-wave study of Chinese adolescents, Wang et al. (2023) found that the common Dark Triad core and Machiavellianism were associated with subsequent suicidal ideation through social alienation. More recently, a three-wave prospective study of university students found that Machiavellianism and psychopathy were positively associated with suicidal ideation through thwarted belongingness and perceived burdensomeness, whereas narcissism showed a partly inverse interpersonal pattern (Huang et al., 2026). Earlier work has also linked sadistic and self-defeating personality features with suicidal ideation (Segal et al., 2015). Taken together, the available evidence suggests that dark traits may relate to suicidality through interpersonal and affective processes, but direct evidence from adult psychiatric samples that simultaneously includes all four Dark Tetrad dimensions remains scarce.

Importantly, dark traits should not be assumed to relate uniformly to adverse mental health outcomes. Their associations depend on the specific trait, context, and intervening psychological processes. Recent research suggests that coping strategies may account for part of the association between individual Dark Tetrad traits and mental health (Dinić & Kovačević, 2026), while emotion-regulation strategies may also play an important role in the relationship between Dark Tetrad traits and anxious-depressive symptomatology (Galán et al., 2026). In a recent psychiatric sample, personality traits were likewise linked with clinically relevant outcomes through affective distress, further supporting a process-oriented approach to personality and psychopathology (M. Mamić et al., 2026a). This shifts the clinically relevant question from whether a trait is merely correlated with suicidal ideation toward identifying which potentially modifiable psychological processes may account for that association.

Depression is among the most consistently documented correlates of suicidal ideation and behavior (Franklin et al., 2017; Hawton et al., 2013; Ribeiro et al., 2018). Anxiety is also associated with suicidal thoughts and behaviors (Bentley et al., 2016), while stressful life events and cumulative stress burden may increase suicide risk (Liu & Miller, 2014). However, depression, anxiety, and stress are strongly interrelated, making it important to examine their unique contributions within a common model.

The main research gap is the scarcity of models in adult psychiatric samples that simultaneously distinguish all four Dark Tetrad dimensions and examine depression, anxiety, and stress as parallel intervening processes in relation to suicidal ideation. In addition, it is rarely assessed whether such associations remain after adjustment for basic demographic and clinical characteristics. Sadism is particularly understudied in this context, although its links with antagonism, interpersonal conflict, maladaptive coping, and emotion-regulation difficulties provide a theoretical basis for expecting that its association with suicidal ideation may be indirect and related to psychological distress (Dinić & Kovačević, 2026; Galán et al., 2026; M. Mamić et al., 2026a).

Accordingly, the primary aim of this study was to examine the direct, specific indirect, total indirect, and total associations of Machiavellianism, narcissism, psychopathy, and sadism with suicidal ideation, with depression, anxiety, and stress considered as parallel intervening variables. The secondary aim was to assess the robustness of the findings in a sensitivity model additionally adjusted for age, sex, hospitalization, and diagnostic group.

## 2. Materials and Methods

### 2.1. Study Design and Participants

This cross-sectional study was conducted at General County Hospital Požega from 1 July 2025 to 1 May 2026. Eligible patients were consecutively invited to participate during the study period. Psychiatric diagnoses were obtained from the patients’ medical records. Participants included both hospitalized and non-hospitalized patients. The final analytic sample comprised 210 participants. For the adjusted analyses, diagnostic categories were grouped into five broader groups: neurotic, stress-related, and somatoform disorders (*n* = 105), alcohol use disorders (*n* = 50), depressive disorders (*n* = 30), bipolar affective disorders (*n* = 15), and psychotic disorders (*n* = 10). Neurotic, stress-related, and somatoform disorders served as the reference diagnostic group in the adjusted analysis. No a priori sample-size calculation was performed; the sample therefore reflected all eligible patients consecutively recruited during the predefined study period.

#### 2.1.1. Inclusion Criteria

Participants were eligible if they were aged 18 years or older, had a confirmed psychiatric diagnosis, and were in a sufficiently stable mental and physical condition to participate in the study. Participants also had to be able to understand the purpose and procedures of the study, provide informed consent, independently and reliably complete the study questionnaires, and have sufficient command of Croatian, the language in which the instruments were administered.

#### 2.1.2. Exclusion Criteria

Participants were excluded if they had acute psychiatric disorganization or another condition that prevented valid informed consent or reliable self-report, severe cognitive impairment, acute intoxication, withdrawal delirium, or another acute mental or physical condition that prevented comprehension of the questions or valid completion of the study instruments.

### 2.2. Instruments

#### 2.2.1. Short Dark Tetrad (SD4)

The Short Dark Tetrad (SD4) was developed as a brief measure of four socially aversive personality traits (Paulhus et al., 2021). The Croatian version of the SD4 has been psychometrically evaluated, with findings supporting its validity for the assessment of the four Dark Tetrad dimensions in the Croatian context (Wertag et al., 2023). The instrument contains 28 items, with seven items assessing each of Machiavellianism, narcissism, psychopathy, and sadism. Responses are provided on a five-point Likert scale ranging from 1 (strongly disagree) to 5 (strongly agree). Each subscale score is obtained by summing the seven relevant items after appropriate coding, yielding a possible range of 7 to 35; higher scores indicate greater expression of the corresponding dark trait. The four dimensions were analyzed separately rather than combined into a total Dark Tetrad score because the aim was to examine trait-specific associations. Internal consistency in the present study was good: McDonald’s ω was 0.875 for Machiavellianism, 0.904 for narcissism, 0.882 for psychopathy, and 0.880 for sadism.

#### 2.2.2. Depression Anxiety Stress Scales-21 (DASS-21)

The DASS-21 contains 21 items divided into three seven-item subscales assessing depression, anxiety, and stress (Henry & Crawford, 2005; Lovibond & Lovibond, 1995). The Croatian adaptation of the DASS-21 has previously demonstrated satisfactory psychometric properties in a heterogeneous psychiatric sample (Ivezić et al., 2012), with additional evidence supporting the reliability and validity of the Croatian version in a clinical population with multiple sclerosis (Rogić Vidaković et al., 2021). Participants rate the extent to which each statement applied to them during the previous week on a four-point scale from 0 to 3. The seven items within each subscale are summed, and in the present study subscale scores were multiplied by two to make them comparable with the original DASS-42 score ranges. Higher scores indicate greater symptom severity. Subscale reliability in the present study was good: McDonald’s ω = 0.863 for depression, ω = 0.863 for anxiety, and ω = 0.867 for stress.

#### 2.2.3. Suicidal Ideation Attributes Scale (SIDAS)

The SIDAS is a brief five-item self-report scale designed to assess the severity of suicidal ideation during the previous month (van Spijker et al., 2014). The SIDAS has demonstrated good psychometric properties in its original validation study and has previously been used in Croatian research examining suicidal ideation (van Spijker et al., 2014; Jovanović et al., 2025). The items assess the frequency of suicidal thoughts, perceived control over them, proximity to a suicide attempt, associated distress, and interference with functioning. Each item is rated on an 11-point scale from 0 to 10. The second item (controllability) is reverse-scored as 10 minus the item response. In accordance with the SIDAS scoring rule, participants reporting no suicidal ideation on the first item received a total score of 0; otherwise, the five scored items were summed. Total scores range from 0 to 50, with higher scores indicating more severe suicidal ideation. In the present sample, the SIDAS demonstrated good internal consistency (McDonald’s ω = 0.859).

The DASS-21 and SIDAS assess overlapping but non-identical reference periods: the DASS-21 refers to symptoms during the previous week, whereas the SIDAS refers to suicidal ideation during the previous month. Accordingly, this temporal mismatch was considered when interpreting the cross-sectional indirect associations and precluded interpretation of the intervening-variable paths as temporally ordered mediation.

### 2.3. Ethical Considerations

The study was conducted in accordance with the Declaration of Helsinki and was approved by the Ethics Committee of General County Hospital Požega (approval No. 02-7/2-2/1-3-2025, 26 June 2025). Participation was voluntary, and informed consent was obtained from all participants before inclusion in the study. Participant confidentiality was maintained during data collection, analysis, and reporting. Because suicidal ideation was directly assessed, its presence was not treated as an automatic exclusion criterion; when clinically relevant suicidal risk was identified, an appropriate clinical assessment and further clinical management were undertaken as indicated. Research procedures did not replace routine psychiatric assessment or treatment and were conducted in accordance with the ethical principles applicable to research involving human participants.

### 2.4. Statistical Analysis

Participant characteristics were summarized using *n* (%) for categorical variables and M (SD) for age. Descriptive analysis of the principal study variables included arithmetic means, standard deviations, medians, quartiles, and ranges. Internal consistency was assessed using McDonald’s ω for the DASS-21, SIDAS, and SD4 subscales (Dunn et al., 2014; McDonald, 1999). Bivariate associations among the main constructs were estimated using Pearson correlation coefficients, with Spearman rank correlations used as a sensitivity analysis. Participants with questionnaires insufficiently completed to permit valid scale scoring were excluded before analysis, and no missing-data imputation was applied.

The primary multivariable analysis was specified as a parallel indirect-effects model in which the four SD4 dimensions were entered simultaneously as exogenous predictors, depression, anxiety, and stress were specified as parallel intervening variables, and the SIDAS total score was the continuous outcome. Direct effects of the Dark Tetrad traits on SIDAS, paths from the Dark Tetrad traits to each DASS dimension, paths from the DASS dimensions to SIDAS, 12 specific indirect effects, four total indirect effects, and four total effects were estimated. DASS residual covariances were freely estimated. The primary model was estimated without covariates. A sensitivity model was subsequently estimated using the same model specification with additional adjustment for age, sex, hospitalization, and diagnostic group. Age was entered as a centered continuous covariate, whereas sex, hospitalization, and diagnosis were dummy-coded. Neurotic, stress-related, and somatoform disorders served as the reference diagnostic category.

Robust maximum likelihood estimation (MLR) was used to estimate regression paths. Indirect and total effects were evaluated separately using maximum likelihood estimation (ML) with 10,000 nonparametric bootstrap resamples and percentile 95% bootstrap confidence intervals. For correlations and regression paths, all tests were two-sided and *p* < 0.05 was considered statistically significant. Indirect and total effects were considered statistically significant when the bootstrap confidence interval did not include zero (Hayes & Rockwood, 2017; Preacher & Hayes, 2008; Zhao et al., 2010). Multicollinearity was assessed using the generalized variance inflation factor (GVIF), adjusted GVIF, approximate tolerance, and condition indices. Potentially influential observations were evaluated using standardized residuals and Cook’s distance, with the conservative 4/*n* criterion additionally examined as a screening threshold. An auxiliary multiple regression representation of the adjusted SIDAS model was used for residual and heteroskedasticity diagnostics. Because the study was cross-sectional, indirect effects were interpreted as statistical associations rather than evidence of causal mediation (Maxwell & Cole, 2007; Maxwell et al., 2011). Both path models were saturated (df = 0); therefore, global fit indices were not interpreted as indicators of model quality.

Because 92 participants (43.8%) had a SIDAS total score of 0, an additional two-part sensitivity analysis was performed to address the substantial floor concentration. In the first part, the occurrence of suicidal ideation was modeled as SIDAS = 0 versus SIDAS > 0 using logistic regression with HC3 robust standard errors. In the second part, SIDAS severity was analyzed only among participants with SIDAS > 0 (*n* = 118) using linear regression with HC3 robust standard errors. The primary and covariate-adjusted two-part models mirrored the corresponding predictor sets used in the main analyses. A two-part approach was selected because SIDAS is a bounded psychometric sum score and a score of zero is a valid observed value rather than a censored observation. A further robustness analysis re-estimated the covariate-adjusted parallel indirect-effects model after excluding the two smallest diagnostic groups, psychotic disorders (*n* = 10) and bipolar affective disorders (*n* = 15), leaving N = 185. MLR was again used for regression paths and 10,000 percentile bootstrap resamples were used for indirect and total effects. Finally, because no a priori sample-size calculation had been performed, a sensitivity power analysis was conducted to characterize the smallest effects detectable with the available sample. For the adjusted SIDAS equation at N = 210, 80% power corresponded to a minimum detectable local effect of f^2^ = 0.040 (partial r = 0.197), whereas 90% power corresponded to f^2^ = 0.054 (partial r = 0.226).

All statistical analyses were performed using R version 4.5.2 (R Foundation for Statistical Computing, Vienna, Austria) in RStudio version 2026.07.1+147 (Posit PBC, Boston, MA, USA). Analyses used readxl for data import, psych for reliability and correlation analyses, lavaan for the parallel indirect-effects models, car for multicollinearity diagnostics, and lmtest for heteroskedasticity diagnostics; base R functions were additionally used for descriptive statistics, residual diagnostics, condition indices, and Cook’s distance. The additional sensitivity analyses used sandwich and lmtest for HC3 robust inference and pwr for sensitivity power calculations.

## 3. Results

### 3.1. Participant Characteristics

A total of 256 eligible patients were consecutively invited to participate. Of these, 27 declined participation. Among the 229 patients who agreed to participate, 19 were excluded from the analysis because their questionnaires were insufficiently completed to permit valid analysis. The final analytic sample therefore consisted of 210 participants, corresponding to 82.0% of all eligible patients approached. The mean age was 42.742 years (SD = 12.194), and 109 participants (51.9%) were women. The largest diagnostic group comprised neurotic, stress-related, and somatoform disorders (105 participants; 50.0%), while 88 participants (41.9%) were hospitalized (Table 1).

### 3.2. Descriptive Statistics

Among the DASS dimensions, the highest median was observed for stress (Mdn = 20), whereas depression and anxiety both had a median of 18. The median suicidal ideation score was 10 (Q1 = 0; Q3 = 31). A total of 92 participants (43.8%) had a SIDAS score of 0, indicating a pronounced floor concentration in the SIDAS distribution (Table 2).

### 3.3. Correlations Among the Main Constructs

All three DASS dimensions were statistically significantly associated with suicidal ideation, with the strongest association observed for depression (r = 0.560; *p* < 0.001), followed by stress (r = 0.509; *p* < 0.001) and anxiety (r = 0.466; *p* < 0.001). Among the dark traits, sadism showed the strongest association with stress (r = 0.402; *p* < 0.001), whereas its association with suicidal ideation was weaker (r = 0.231; *p* < 0.001). The DASS dimensions were moderately to strongly intercorrelated (r = 0.611–0.653). Spearman sensitivity correlations were highly similar in magnitude (ρ = 0.554 for depression, ρ = 0.505 for stress, and ρ = 0.467 for anxiety with SIDAS; all *p* < 0.001). The only inferential difference was a small narcissism–SIDAS association that reached nominal significance with Spearman correlation (ρ = 0.143; *p* = 0.039), whereas the corresponding Pearson correlation did not (r = 0.135; *p* = 0.051) (Table 3).

### 3.4. Primary Parallel Indirect-Effects Model

In the primary model without covariates, the Dark Tetrad traits and DASS dimensions jointly explained 36.3% of the variance in suicidal ideation. Explained variance was 11.6% for depression, 12.8% for anxiety, and 18.7% for stress (Table 4).

Among the Dark Tetrad traits, sadism showed the clearest associations with the DASS dimensions. Higher sadism was associated with higher anxiety (β = 0.267; *p* < 0.001) and higher stress (β = 0.286; *p* < 0.001). The association between sadism and depression did not reach statistical significance (β = 0.169; *p* = 0.051), and the association between psychopathy and stress was likewise not statistically significant after robust estimation (β = 0.163; *p* = 0.056). When depression, anxiety, and stress were entered simultaneously in the SIDAS equation, depression showed the strongest independent association with suicidal ideation (β = 0.368; *p* < 0.001), followed by stress (β = 0.230; *p* = 0.004). Anxiety did not make a statistically significant unique contribution. None of the four Dark Tetrad traits had a statistically significant direct association with SIDAS in the primary model (Table 5).

### 3.5. Indirect and Total Effects in the Primary Model

**Figure 1:**
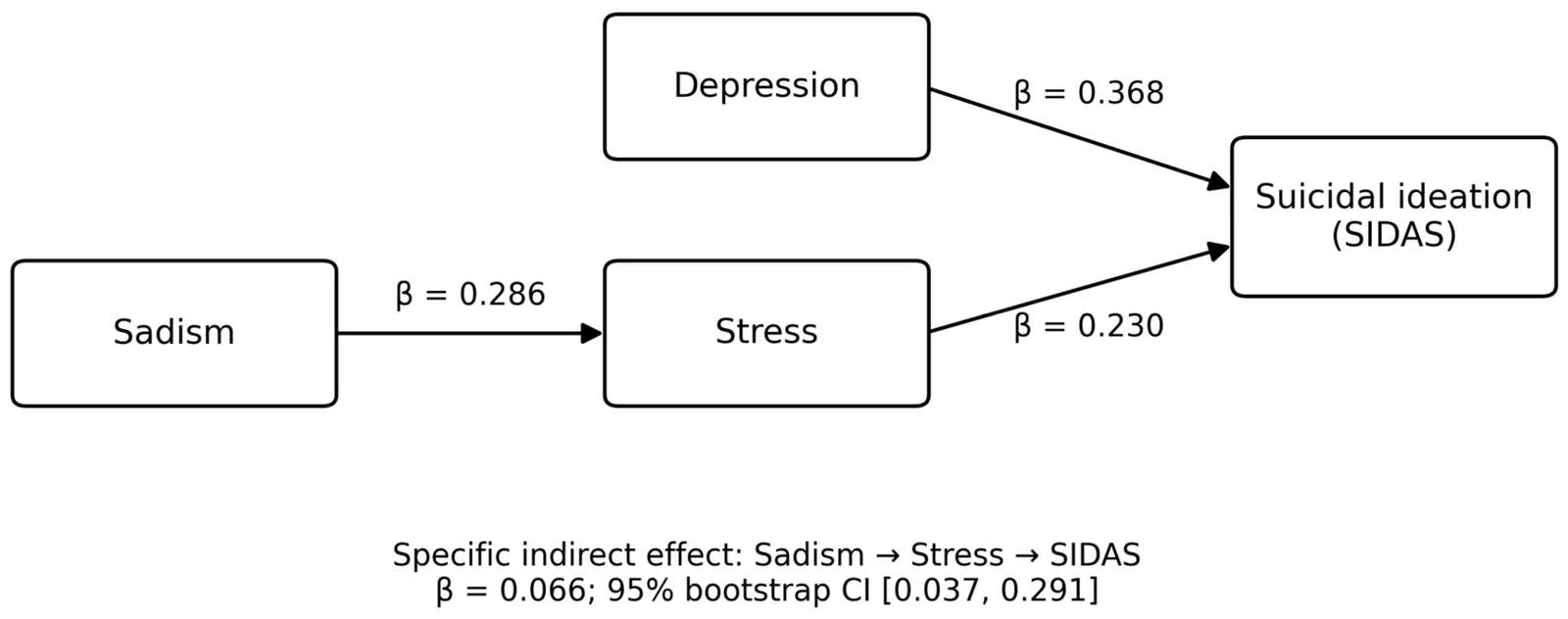
Key statistically significant standardized paths in the primary parallel indirect-effects model. Values are standardized β coefficients. Only the key statistically significant paths are shown for visual clarity. The specific indirect effect of sadism through stress was β = 0.066 (95% bootstrap CI [0.037, 0.291]).

Bootstrap analysis with 10,000 resamples showed that the only statistically significant specific indirect pathway in the primary model was sadism → stress → suicidal ideation (B = 0.147; SE = 0.065; β = 0.066; 95% bootstrap CI [0.037, 0.291]). The indirect pathways from sadism through depression and anxiety were not statistically significant because their confidence intervals included zero. None of the specific indirect effects of Machiavellianism, narcissism, or psychopathy had a bootstrap confidence interval that excluded zero (Figure 1 and Table 6).

The total indirect effect of sadism through depression, anxiety, and stress was statistically significant (B = 0.327; SE = 0.122; β = 0.147; 95% bootstrap CI [0.098, 0.576]). In contrast, the total effect of sadism on suicidal ideation was not statistically significant because its bootstrap confidence interval included zero (B = 0.300; β = 0.134; 95% bootstrap CI [−0.089, 0.683]). The total indirect and total effects of Machiavellianism, narcissism, and psychopathy were also not statistically significant (Table 7).

### 3.6. Sensitivity Analysis: Covariate-Adjusted Model

To assess the robustness of the primary findings, the same parallel indirect-effects model was re-estimated with additional adjustment for age, sex, hospitalization, and diagnostic group. The central pattern remained unchanged. Sadism remained associated with anxiety (β = 0.269; *p* = 0.001) and stress (β = 0.294; *p* < 0.001), while its association with depression was not statistically significant (β = 0.175; *p* = 0.0501). Depression (β = 0.315; *p* < 0.001) and stress (β = 0.192; *p* = 0.017) remained independently associated with suicidal ideation. The specific indirect pathway sadism → stress → suicidal ideation remained statistically significant after adjustment (B = 0.126; SE = 0.064; β = 0.057; 95% bootstrap CI [0.016, 0.266]). The total indirect effect of sadism also remained statistically significant (B = 0.308; SE = 0.116; β = 0.138; 95% bootstrap CI [0.088, 0.543]), whereas the total effect of sadism remained non-significant. The adjusted model explained 39.8% of the variance in SIDAS, compared with 36.3% in the primary model (Table 8).

Among the covariates, hospitalization was the only variable that showed a statistically significant independent association with suicidal ideation in the adjusted model. Hospitalized participants had, on average, a 5.737-point higher SIDAS score than non-hospitalized participants after adjustment for all other variables (β = 0.172; *p* = 0.006). Age, sex, and the individual diagnostic contrasts did not show statistically significant independent associations with SIDAS. Neurotic, stress-related, and somatoform disorders were the reference diagnostic category (Table 9).

Model diagnostics did not indicate problematic multicollinearity or highly influential observations. The maximum adjusted GVIF was 1.498, the minimum approximate tolerance was 0.446, and the maximum condition index was 3.452. No standardized residual exceeded ±3 (maximum absolute standardized residual = 2.509), and the maximum Cook’s distance was 0.042. Twelve observations exceeded the conservative 4/*n* screening threshold, but none had Cook’s distance greater than 1. Residuals from the auxiliary adjusted SIDAS regression did not significantly deviate from normality (Shapiro–Wilk W = 0.990; *p* = 0.156). The Breusch–Pagan test indicated heteroskedasticity (BP = 28.177; df = 14; *p* = 0.013), supporting the use of robust MLR inference for the regression paths. No observation was excluded solely on the basis of influence diagnostics.

### 3.7. Two-Part Sensitivity Analysis of SIDAS

The two-part sensitivity analysis separated the occurrence of any suicidal ideation (SIDAS = 0 vs. SIDAS > 0) from its severity among participants with SIDAS > 0. In the covariate-adjusted logistic model, depression was significantly associated with the occurrence of suicidal ideation (OR = 1.068; 95% CI [1.023, 1.114]; *p* = 0.003), and hospitalization was also associated with higher odds of SIDAS > 0 (OR = 2.399; 95% CI [1.106, 5.202]; *p* = 0.027). Anxiety, stress, and the four Dark Tetrad traits were not significantly associated with occurrence after simultaneous adjustment. Among the 118 participants with SIDAS > 0, stress was significantly associated with greater SIDAS severity (B = 0.287; robust SE = 0.126; 95% CI [0.039, 0.534]; *p* = 0.025). Sadism was also positively associated with severity in this restricted positive-score subsample (B = 0.414; robust SE = 0.198; 95% CI [0.026, 0.802]; *p* = 0.039), whereas depression and anxiety were not statistically significant. These findings indicate that the occurrence and severity components of SIDAS showed partly distinct association patterns (Table 10).

### 3.8. Restricted Diagnostic-Group Sensitivity Analysis

To determine whether the two smallest diagnostic subgroups materially influenced the covariate-adjusted findings, the model was re-estimated after excluding participants with psychotic disorders (*n* = 10) and bipolar affective disorders (*n* = 15), leaving N = 185. The central pattern was preserved. Sadism remained associated with stress (β = 0.283; *p* < 0.001), depression remained independently associated with SIDAS (β = 0.333; *p* < 0.001), and stress remained independently associated with SIDAS (β = 0.185; *p* = 0.031). The direct sadism–SIDAS path remained null (β = 0.004; *p* = 0.959). Importantly, the specific indirect effect sadism → stress → SIDAS remained statistically significant (B = 0.115; β = 0.052; 95% bootstrap CI [0.006, 0.259]). The restricted model explained 38.9% of the variance in SIDAS, closely matching the 39.8% explained by the full covariate-adjusted model (Table 11).

### 3.9. Sensitivity Power Analysis

The sensitivity power analysis indicated that, for a single path in the covariate-adjusted SIDAS equation at N = 210, the minimum detectable local effect was f^2^ = 0.040 (partial R^2^ = 0.039; partial r = 0.197) at 80% power and f^2^ = 0.054 (partial R^2^ = 0.051; partial r = 0.226) at 90% power. For the overall adjusted regression equation, the corresponding minimum detectable effects were f^2^ = 0.093 at 80% power and f^2^ = 0.116 at 90% power. Thus, the available sample was reasonably sensitive to small-to-moderate effects, while very small local effects could have remained undetected.

## 4. Discussion

The main finding of this study is that the association between Dark Tetrad traits and suicidal ideation was not adequately characterized by simple direct effects alone. Sadism showed the clearest trait-specific pattern: it was associated with both anxiety and stress, but only stress retained an independent association with suicidal ideation when the DASS dimensions were modeled simultaneously; accordingly, the specific indirect effect of sadism through stress was supported in the primary model. Importantly, this specific indirect association remained statistically significant in the sensitivity model after adjustment for age, sex, hospitalization, and diagnostic group (B = 0.126; 95% bootstrap CI [0.016, 0.266]). Thus, the central pattern was robust to adjustment for the basic demographic and clinical covariates included in the analysis. Because the data are cross-sectional, however, this pathway should be interpreted as a statistical indirect association rather than evidence that sadism causally increases stress or that stress subsequently causes suicidal ideation. The additional sensitivity analyses strengthened this interpretation: the key sadism → stress → SIDAS indirect association remained significant after excluding the two smallest diagnostic groups, and the two-part analysis showed distinct association patterns for the occurrence and severity components of SIDAS.

The bivariate findings help contextualize the multivariable model. Among the DASS dimensions, sadism was most strongly associated with stress (r = 0.402), whereas its bivariate association with suicidal ideation was weaker (r = 0.231). This pattern is compatible with the possibility that the clinical relevance of a relatively stable personality characteristic may be expressed more strongly through proximal emotional distress than through a direct association with suicidal ideation. At the same time, the substantial correlations among the DASS dimensions indicate a shared negative-affect component, while their differing contributions to SIDAS suggest that modeling them separately can still provide clinically informative distinctions.

The two-part analysis further clarified the clinical structure of the outcome. Depression was associated primarily with the occurrence of suicidal ideation, whereas stress was associated with its severity among participants who reported suicidal ideation. Sadism was also associated with severity within the SIDAS-positive subgroup, although this secondary finding should be interpreted cautiously because it was estimated in only 118 participants and was not reflected as a significant direct sadism–SIDAS path in the full-sample model. This distinction reinforces the broader possibility that different clinical processes may be associated with the occurrence and the severity of suicidal ideation.

The findings are broadly consistent with the limited literature linking dark personality traits to internalizing outcomes. Previous longitudinal work in adolescents suggested that social alienation may account for part of the association between the common Dark Triad core and suicidal ideation (Wang et al., 2023), and earlier findings linked sadistic and self-defeating personality features with suicidal ideation in older adults (Segal et al., 2015). More recent studies have highlighted coping strategies and emotion regulation as potential mechanisms connecting Dark Tetrad traits with mental health outcomes (Dinić & Kovačević, 2026; Galán et al., 2026). In addition, recent work in psychiatric patients has shown that affective distress can operate as an intervening process linking personality characteristics with clinically relevant behavioral outcomes (M. Mamić et al., 2026a). The present results extend this line of inquiry to suicidal ideation and suggest that stress may be particularly relevant when sadism is considered alongside the other Dark Tetrad traits and the overlapping dimensions of depression and anxiety.

The stress-diathesis framework provides a useful interpretation of this pattern (Mann et al., 1999). Dark traits may be conceptualized as relatively stable dispositional characteristics, whereas DASS dimensions capture more temporally proximal emotional states. From this perspective, sadism need not directly generate suicidal ideation. Its relevance may instead emerge through recurrent interpersonal conflict, frustration, hostile interactions, or less adaptive coping patterns that contribute to sustained tension and stress. This interpretation also accords with broader conceptualizations of suicide risk that emphasize the interaction between enduring vulnerability and fluctuating states of acute distress, including the concepts of psychache and fluid vulnerability (Bryan et al., 2020; Shneidman, 1993). Consistent with this process-oriented view, recent cross-sectional work among nurses found that perceived stress was associated with quality of life partly through burnout, illustrating how stress-related associations may operate through modifiable psychological processes (M. Mamić et al., 2026b).

The IMV model offers an additional, although not directly tested, framework (O’Connor & Kirtley, 2018). It proposes that background factors and vulnerabilities create the context in which motivational processes contribute to suicidal ideation, followed by volitional factors that influence the transition from ideation to behavior. Within such a framework, sadism could be positioned as a more distal dispositional characteristic and stress as a more proximal affective state. However, DASS stress is not equivalent to defeat, entrapment, humiliation, or other constructs that have specific roles in the IMV model. Future studies should therefore directly measure these constructs and evaluate whether interpersonal problems, defeat, entrapment, and stress form sequential pathways linking dark traits to suicidal ideation.

Interpersonal models remain relevant because antagonistic personality characteristics may contribute to conflict, social disconnection, or alienation, which may in turn increase psychological distress (Van Orden et al., 2010; Wang et al., 2023). Recent Croatian SIDAS research similarly linked suicidal thoughts with loneliness and related psychosocial characteristics in a non-psychiatric sample (Jovanović et al., 2025). These observations support future longitudinal models that directly test interpersonal problems or alienation as antecedents of stress and suicidal ideation rather than assuming such mechanisms from the present cross-sectional data.

Sadism requires cautious interpretation. The primary and covariate-adjusted models did not identify a significant direct sadism–SIDAS path, whereas bootstrap analyses consistently supported the specific indirect association through stress. The two-part analysis additionally identified a positive association between sadism and SIDAS severity among participants with SIDAS > 0, but not with the occurrence of suicidal ideation. Taken together, these findings do not support treating sadism as a stand-alone suicide-risk factor; rather, they suggest that its possible clinical relevance may depend on stress-related and severity-specific processes.

Depression showed the strongest independent association with suicidal ideation, which is consistent with extensive evidence linking depressive symptoms and hopelessness to suicidal thoughts and behaviors (Franklin et al., 2017; Hawton et al., 2013; Ribeiro et al., 2018). Nevertheless, meta-analytic evidence indicates that individual risk factors have limited ability to predict suicidal outcomes at the individual level (Franklin et al., 2017). Accordingly, neither DASS depression nor any Dark Tetrad trait should be used as a stand-alone clinical screening tool for suicide risk. Their value, if any, lies in contributing to a broader clinical formulation together with current symptoms, history of suicidal behavior, psychosocial stressors, protective factors, and clinical judgment.

Stress retained a significant contribution to SIDAS even after depression and anxiety were entered simultaneously. DASS stress captures tension, irritability, difficulty relaxing, and a tendency toward overreaction (Henry & Crawford, 2005; Lovibond & Lovibond, 1995). In a psychiatric population, this continuous distress may therefore provide clinically relevant information beyond diagnostic category alone. Anxiety, by contrast, was moderately correlated with suicidal ideation but did not retain a significant unique contribution after depression and stress were included. This is not contradictory: because the DASS dimensions are strongly correlated, part of the bivariate association between anxiety and SIDAS is shared with depression and stress. The model diagnostics did not indicate problematic multicollinearity, suggesting that this pattern is more likely to reflect shared variance in negative affect than numerical instability.

Hospitalization was independently associated with higher suicidal ideation after adjustment for the other variables. Hospitalized participants had an approximately 5.74-point higher SIDAS score than non-hospitalized participants, which may reflect greater clinical severity, acuity, or complexity among patients requiring inpatient care. Hospitalization itself should not be interpreted as a causal determinant of suicidal ideation because admission is likely to be influenced by the same clinical characteristics that are related to suicide risk. Future research should therefore include more detailed indicators such as previous suicide attempts, history of self-harm, number and duration of psychiatric hospitalizations, illness duration, current pharmacotherapy, and markers of acute clinical severity.

The clinical implications of the findings concern assessment of processes rather than labeling personality. Dark Tetrad scores should not be used in isolation to classify patients as suicidal or non-suicidal, and the present results do not justify stigmatizing interpretations of patients with elevated antagonistic traits. A more appropriate application would be to consider whether recurrent interpersonal conflict, hostility, maladaptive coping, emotion-regulation difficulties, and sustained stress contribute to an individual patient’s clinical formulation. This is particularly relevant because personality traits are relatively stable, whereas stress, coping strategies, and emotion-regulation processes are more modifiable. The findings therefore support further investigation of proximal processes that may represent clinically actionable targets rather than attempting to change the personality trait itself. Future intervention studies could therefore test whether structured stress-management, emotion-regulation, and interpersonal coping interventions reduce suicidal ideation severity among patients with elevated antagonistic traits, while avoiding the use of personality scores as stand-alone risk classifiers. This intervention-oriented perspective is also compatible with findings from a psychiatric day-hospital sample in which interdisciplinary treatment was accompanied by a reduction in neurotic defense mechanisms among patients with anxiety and/or depressive disorders (M. Mamić, 2026).

Several methodological features strengthen the study. All four Dark Tetrad dimensions were examined simultaneously, which reduces the risk of attributing to one trait an association that is shared with the others. Depression, anxiety, and stress were also modeled in parallel, allowing their unique contributions to be considered within the same model. A covariate-adjusted sensitivity analysis was used to assess the robustness of the primary findings. Regression paths were evaluated using robust MLR inference, while indirect and total effects were evaluated using percentile bootstrap confidence intervals based on 10,000 resamples. Spearman sensitivity correlations closely reproduced the principal bivariate pattern, and influence diagnostics did not indicate highly influential observations. These features increase confidence in the internal statistical coherence of the observed pattern, while not overcoming the limitations inherent to a cross-sectional observational design. In addition, a two-part analysis directly addressed the 43.8% SIDAS floor concentration, and re-estimation after excluding the psychotic and bipolar groups showed that the main indirect effect was not driven by the smallest diagnostic subgroups.

Several limitations should be considered. First, the cross-sectional design does not establish temporal ordering. Although the model statistically decomposes direct and indirect associations, it cannot demonstrate that sadism precedes stress or that stress subsequently causes suicidal ideation (Maxwell & Cole, 2007; Maxwell et al., 2011); reverse and reciprocal relationships remain possible. Second, all principal constructs were assessed using self-report instruments, which may introduce shared-method variance, response bias, and socially desirable responding, particularly for socially aversive personality traits. Third, the measures cover different reference periods: the DASS-21 assesses symptoms during the previous week, whereas the SIDAS assesses suicidal ideation during the previous month. This temporal mismatch further limits mechanistic interpretation of the indirect pathways. The SIDAS distribution also showed a substantial floor concentration, with 43.8% of participants scoring zero. Although robust estimation and sensitivity analyses supported the principal pattern, this concentration may reduce discrimination among participants at the lower end of suicidal ideation severity. Although the two-part analysis addressed the accumulation of zero SIDAS scores, it does not establish temporal ordering or convert the cross-sectional indirect effect into causal mediation.

Fourth, this was a single-center psychiatric sample, which may limit generalizability to other hospitals, community settings, and cultural contexts. The diagnostic groups were broad and uneven in size, and statistical power and precision were particularly limited for the psychotic (*n* = 10) and bipolar (*n* = 15) contrasts. Although excluding these groups did not materially change the central results, disorder-specific conclusions should not be drawn from these small subgroups. The sensitivity power analysis also indicated that very small local effects could have remained undetected; consequently, null or marginal paths, such as the sadism–depression association, should not be interpreted as proof of no association. Fifth, the inclusion criteria required participants to be sufficiently stable to provide informed consent and complete self-report measures reliably. Consequently, the most acutely disorganized, cognitively impaired, intoxicated, or medically unstable patients were not represented, and the findings may not generalize to the most acute psychiatric presentations. Sixth, the model did not include several established or clinically relevant suicide-risk variables, including previous suicide attempts, self-harm history, hopelessness, defeat and entrapment, social support, trauma, impulsivity, aggression, current pharmacotherapy, and illness duration. Residual confounding by these unmeasured factors is therefore possible.

Future research should evaluate the proposed indirect-effects model using prospective longitudinal designs that can establish temporal precedence more convincingly. Ideally, Dark Tetrad traits, depression, anxiety, stress, and suicidal ideation should be assessed repeatedly across at least three measurement occasions, allowing researchers to determine whether Dark Tetrad traits predict subsequent changes in emotional distress and whether these changes, in turn, predict later suicidal ideation. Such designs would also permit examination of reciprocal effects, including the possibility that suicidal ideation or psychological distress may influence later perceptions of stress. Cross-lagged panel models, random-intercept cross-lagged models, or longitudinal mediation models could help distinguish stable between-person differences from within-person changes over time. Larger multicenter samples would additionally allow the stability of the sadism–stress–suicidal ideation pathway to be tested across diagnostic groups and treatment settings. Repeated assessment of stress and suicidal ideation at shorter intervals may be particularly informative because these constructs can fluctuate substantially over time, whereas Dark Tetrad traits are expected to remain comparatively stable. Ecological momentary assessment could additionally be used to examine short-term fluctuations in stress and suicidal thoughts, while serial longitudinal models incorporating interpersonal conflict, social alienation, defeat, entrapment, coping, and emotion regulation could clarify whether these processes further account for the observed indirect association.

## 5. Conclusions

In this psychiatric sample, none of the Dark Tetrad traits showed a statistically significant direct association with suicidal ideation in the primary model, and this pattern remained unchanged after adjustment for age, sex, hospitalization, and diagnostic group. Sadism emerged as the trait most consistently associated with stress, and the specific indirect pathway sadism → stress → suicidal ideation was statistically significant in the primary model, remained significant in the covariate-adjusted model, and was preserved after excluding the two smallest diagnostic groups. Two-part sensitivity analyses further indicated that depression was associated with the occurrence of suicidal ideation, whereas stress was associated with its severity among participants with SIDAS > 0; sadism also showed a severity-specific association in this secondary analysis. These findings support a process-oriented interpretation in which proximal emotional distress may be more clinically informative than dark personality traits alone. Because the study was cross-sectional, the indirect pathways should not be interpreted causally; replication in independent samples and longitudinal research is needed to clarify temporal and mechanistic relationships.

## Figures and Tables

**Table 1:** Sociodemographic and clinical characteristics of participants.

		*n* (%)
Sex	Male	101 (48.1)
	Female	109 (51.9)
Education level	Primary	42 (20.0)
	Secondary	101 (48.1)
	Higher/University	67 (31.9)
Employment status	Unemployed	117 (55.7)
	Retired	8 (3.8)
	Employed	85 (40.5)
Diagnosis	Bipolar affective disorders	15 (7.1)
	Depressive disorders	30 (14.3)
	Neurotic, stress-related, and somatoform disorders	105 (50.0)
	Alcohol use disorders	50 (23.8)
	Psychotic disorders	10 (4.8)
Hospitalization	Yes	88 (41.9)
	No	122 (58.1)
		M (SD)
Age, years		42.742 (12.194)

Note: *n*—number of participants; %—percentage; M—arithmetic mean; SD—standard deviation.

**Table 2:** Descriptive statistics for the main variables.

	M	SD	Mdn	Q1	Q3	Min	Max
Suicidal ideation (SIDAS)	15.505	16.455	10	0.000	31.000	0	50
Depression (DASS-21)	19.171	11.922	18	10.000	30.000	0	42
Anxiety (DASS-21)	19.600	11.854	18	10.000	28.000	0	42
Stress (DASS-21)	19.429	12.031	20	10.000	30.000	0	42
Machiavellianism	19.510	6.928	20	13.250	25.000	7	34
Narcissism	20.514	7.122	21	15.000	25.750	7	35
Psychopathy	20.662	7.938	21	14.000	27.000	7	35
Sadism	20.386	7.367	20	15.000	26.000	7	35

Note: M—arithmetic mean; SD—standard deviation; Mdn—median; Q1—first quartile; Q3—third quartile; Min—minimum value; Max—maximum value.

**Table 3:** Pearson correlations among the main constructs.

	1	2	3	4	5	6	7	8
1. Machiavellianism	—							
2. Narcissism	0.475 **	—						
3. Psychopathy	0.533 **	0.425 **	—					
4. Sadism	0.501 **	0.454 **	0.528 **	—				
5. Depression	0.284 **	0.210 *	0.253 **	0.295 **	—			
6. Anxiety	0.256 **	0.114	0.246 **	0.327 **	0.650 **	—		
7. Stress	0.274 **	0.248 **	0.344 **	0.402 **	0.611 **	0.653 **	—	
8. Suicidal ideation	0.201 *	0.135	0.231 **	0.231 **	0.560 **	0.466 **	0.509 **	—

Note: r—Pearson correlation coefficient; *p*—statistical significance; * *p* < 0.01; ** *p* < 0.001. 1—Machiavellianism; 2—Narcissism; 3—Psychopathy; 4—Sadism; 5—Depression; 6—Anxiety; 7—Stress; 8—Suicidal ideation.

**Table 4:** Explained variance of endogenous variables in the primary model.

	R^2^	Explained Variance (%)
Depression	0.116	11.6
Anxiety	0.128	12.8
Stress	0.187	18.7
Suicidal ideation	0.363	36.3

Note: R^2^—coefficient of determination.

**Table 5:** Regression paths in the primary parallel indirect-effects model.

Outcome	Predictor	B	SE	β	*p*	95% CI
Depression	Machiavellianism	0.248	0.144	0.144	0.084	[−0.034, 0.529]
Depression	Narcissism	0.058	0.128	0.035	0.651	[−0.193, 0.310]
Depression	Psychopathy	0.108	0.142	0.072	0.446	[−0.170, 0.386]
Depression	Sadism	0.274	0.140	0.169	0.051	[−0.001, 0.549]
Anxiety	Machiavellianism	0.219	0.149	0.128	0.140	[−0.072, 0.510]
Anxiety	Narcissism	−0.170	0.125	−0.102	0.173	[−0.416, 0.075]
Anxiety	Psychopathy	0.120	0.131	0.080	0.361	[−0.137, 0.377]
Anxiety	Sadism	0.429	0.130	0.267	<0.001	[0.175, 0.684]
Stress	Machiavellianism	0.046	0.144	0.027	0.748	[−0.236, 0.329]
Stress	Narcissism	0.062	0.131	0.036	0.638	[−0.195, 0.318]
Stress	Psychopathy	0.247	0.130	0.163	0.056	[−0.007, 0.501]
Stress	Sadism	0.467	0.130	0.286	<0.001	[0.212, 0.722]
Suicidal ideation	Depression	0.508	0.116	0.368	<0.001	[0.280, 0.736]
Suicidal ideation	Anxiety	0.095	0.129	0.068	0.463	[−0.159, 0.348]
Suicidal ideation	Stress	0.315	0.109	0.230	0.004	[0.101, 0.529]
Suicidal ideation	Machiavellianism	0.013	0.171	0.005	0.941	[−0.322, 0.348]
Suicidal ideation	Narcissism	−0.068	0.164	−0.030	0.677	[−0.389, 0.253]
Suicidal ideation	Psychopathy	0.120	0.154	0.058	0.434	[−0.181, 0.422]
Suicidal ideation	Sadism	−0.027	0.164	−0.012	0.869	[−0.349, 0.295]

Note: B—unstandardized regression coefficient; SE—robust standard error; β—standardized regression coefficient; *p*—statistical significance based on MLR; CI—confidence interval; DASS—Depression Anxiety Stress Scales; SIDAS—Suicidal Ideation Attributes Scale; SD4—Short Dark Tetrad.

**Table 6:** Specific indirect effects in the primary model.

Indirect Pathway	B	SE	β	95% Bootstrap CI
Machiavellianism → depression → SIDAS	0.126	0.080	0.053	[−0.018, 0.297]
Machiavellianism → anxiety → SIDAS	0.021	0.039	0.009	[−0.042, 0.118]
Machiavellianism → stress → SIDAS	0.015	0.049	0.006	[−0.077, 0.124]
Narcissism → depression → SIDAS	0.030	0.068	0.013	[−0.100, 0.169]
Narcissism → anxiety → SIDAS	−0.016	0.031	−0.007	[−0.094, 0.035]
Narcissism → stress → SIDAS	0.019	0.045	0.008	[−0.065, 0.116]
Psychopathy → depression → SIDAS	0.055	0.077	0.026	[−0.091, 0.214]
Psychopathy → anxiety → SIDAS	0.011	0.026	0.005	[−0.039, 0.070]
Psychopathy → stress → SIDAS	0.078	0.050	0.038	[−0.003, 0.189]
Sadism → depression → SIDAS	0.139	0.085	0.062	[−0.004, 0.329]
Sadism → anxiety → SIDAS	0.041	0.062	0.018	[−0.076, 0.174]
Sadism → stress → SIDAS	0.147	0.065	0.066	[0.037, 0.291]

Note: B—unstandardized indirect effect; SE—bootstrap standard error; β—standardized indirect effect; CI—confidence interval; SIDAS—Suicidal Ideation Attributes Scale. Percentile 95% bootstrap confidence intervals based on 10,000 resamples are shown. An indirect effect was considered statistically significant when the confidence interval did not include zero.

**Table 7:** Total indirect and total effects of Dark Tetrad traits in the primary model.

Trait	Effect	B	SE	β	95% Bootstrap CI
Machiavellianism	Total indirect effect	0.161	0.120	0.068	[−0.069, 0.407]
Machiavellianism	Total effect	0.174	0.210	0.073	[−0.240, 0.585]
Narcissism	Total indirect effect	0.033	0.109	0.014	[−0.178, 0.252]
Narcissism	Total effect	−0.036	0.195	−0.015	[−0.413, 0.351]
Psychopathy	Total indirect effect	0.144	0.115	0.069	[−0.086, 0.367]
Psychopathy	Total effect	0.264	0.190	0.128	[−0.106, 0.634]
Sadism	Total indirect effect	0.327	0.122	0.147	[0.098, 0.576]
Sadism	Total effect	0.300	0.196	0.134	[−0.089, 0.683]

Note: B—unstandardized effect; SE—bootstrap standard error; β—standardized effect; CI—confidence interval. Percentile 95% bootstrap confidence intervals based on 10,000 resamples are shown. Statistical significance was determined by whether the confidence interval excluded zero.

**Table 8:** Comparison of key findings in the primary and covariate-adjusted models.

Effect/Outcome	Primary B	Primary β	Primary *p*/95% CI	Adjusted B	Adjusted β	Adjusted *p*/95% CI
Sadism → anxiety	0.429	0.267	*p* = 0.001	0.432	0.269	*p* = 0.001
Sadism → stress	0.467	0.286	*p* < 0.001	0.480	0.294	*p* < 0.001
Depression → SIDAS	0.508	0.368	*p* < 0.001	0.435	0.315	*p* < 0.001
Stress → SIDAS	0.315	0.230	*p* = 0.004	0.263	0.192	*p* = 0.017
Sadism → stress → SIDAS	0.147	0.066	CI [0.037, 0.291]	0.126	0.057	CI [0.016, 0.266]
Total indirect effect of sadism	0.327	0.147	CI [0.098, 0.576]	0.308	0.138	CI [0.088, 0.543]
Total effect of sadism	0.300	0.134	CI [−0.089, 0.683]	0.333	0.149	CI [−0.041, 0.695]
R^2^ for SIDAS	0.363	—	36.3%	0.398	—	39.8%

Note: B—unstandardized regression coefficient or effect; β—standardized regression coefficient or effect; *p*—statistical significance; CI—confidence interval; R^2^—coefficient of determination; SIDAS—Suicidal Ideation Attributes Scale. *p* values for regression paths are based on robust MLR inference. Confidence intervals for indirect and total effects are percentile 95% bootstrap confidence intervals based on 10,000 resamples.

**Table 9:** Associations of covariates with suicidal ideation in the covariate-adjusted model.

Covariate	B	SE	β	*p*	95% CI
Age	0.048	0.073	0.035	0.512	[−0.095, 0.190]
Female sex	1.444	1.737	0.044	0.406	[−1.961, 4.849]
Hospitalization	5.737	2.107	0.172	0.006	[1.608, 9.866]
Bipolar affective disorders	3.718	3.689	0.058	0.314	[−3.513, 10.948]
Depressive disorders	0.623	3.123	0.013	0.842	[−5.499, 6.744]
Alcohol use disorders	2.249	2.187	0.058	0.304	[−2.037, 6.534]
Psychotic disorders	3.726	4.013	0.048	0.353	[−4.139, 11.590]

Note: B—unstandardized regression coefficient; SE—robust standard error; β—standardized regression coefficient; *p*—statistical significance based on MLR; CI—confidence interval; SIDAS—Suicidal Ideation Attributes Scale. Reference categories were male sex, non-hospitalized participants, and neurotic, stress-related, and somatoform disorders.

**Table 10:** Key results of the covariate-adjusted two-part sensitivity analysis.

Predictor	Occurrence: OR	95% CI	*p*	Severity (SIDAS > 0): B	95% CI	*p*
Depression	1.068	[1.023, 1.114]	0.003	0.156	[−0.118, 0.430]	0.268
Anxiety	1.027	[0.983, 1.072]	0.236	0.059	[−0.235, 0.352]	0.696
Stress	1.027	[0.988, 1.068]	0.171	0.287	[0.039, 0.534]	0.025
Machiavellianism	0.985	[0.918, 1.057]	0.674	0.066	[−0.301, 0.433]	0.724
Narcissism	1.036	[0.972, 1.103]	0.276	−0.321	[−0.679, 0.038]	0.082
Psychopathy	1.012	[0.952, 1.077]	0.693	−0.051	[−0.425, 0.322]	0.787
Sadism	0.952	[0.894, 1.015]	0.133	0.414	[0.026, 0.802]	0.039
Hospitalization	2.399	[1.106, 5.202]	0.027	2.854	[−1.642, 7.350]	0.216

Note: OR—odds ratio; B—unstandardized regression coefficient; CI—confidence interval; *p*—statistical significance; SIDAS—Suicidal Ideation Attributes Scale. Occurrence estimates are from logistic regression with HC3 robust standard errors; severity estimates are from linear regression with HC3 robust standard errors among participants with SIDAS > 0. Age, sex, and diagnostic contrasts were included in the adjusted models but are not displayed.

**Table 11:** Key results of the restricted-sample sensitivity analysis (N = 185).

Path/effect	B	SE	β	*p*/95% Bootstrap CI
Sadism → stress	0.464	0.137	0.283	*p* < 0.001
Depression → SIDAS	0.455	0.129	0.333	*p* < 0.001
Stress → SIDAS	0.249	0.115	0.185	*p* = 0.031
Sadism → SIDAS	0.008	0.165	0.004	*p* = 0.959
Sadism → stress → SIDAS	0.115	0.065	0.052	CI [0.006, 0.259]

Note: B—unstandardized regression coefficient or indirect effect; SE—robust standard error for MLR paths and bootstrap standard error for the indirect effect; β—standardized coefficient or effect; *p*—statistical significance; CI—confidence interval; SIDAS—Suicidal Ideation Attributes Scale. The indirect effect is based on 10,000 percentile bootstrap resamples.

## Data Availability

The data are not publicly available because they contain sensitive clinical information. Requests for access may be directed to the corresponding author and are subject to applicable ethical and institutional requirements.
